# Prediction of buckling damage of steel equal angle structural members using hybrid machine learning techniques

**DOI:** 10.1038/s41598-025-87869-w

**Published:** 2025-02-08

**Authors:** Nang Xuan Ho, Tien-Thinh Le, The-Hung Dinh, Van-Hai Nguyen

**Affiliations:** 1 Faculty of Vehicle and Energy Engineering, Phenikaa University, Yen Nghia, Ha Dong, Hanoi, 12116 Vietnam; 2 Faculty of Mechanical Engineering and Mechatronics, Phenikaa University, Yen Nghia, Ha Dong, Hanoi, 12116 Vietnam; 3 Phenikaa Research and Technology Institute (PRATI), A&A Green Phoenix Group JSC, No. 167 Hoang Ngan, Trung Hoa, Hanoi, 11313 Cau Giay Vietnam

**Keywords:** Steel equal angle, Buckling damage, Surrogate model, Artificial neural network, Particle swarm optimization., Civil engineering, Mechanical engineering

## Abstract

This article deals with prediction of buckling damage of steel equal angle structural members using a surrogate model combining machine learning and metaheuristic optimization technique. In particular, a hybrid Artificial Intelligence (AI)-based model involving Artificial Neural Network (ANN) and Particle Swarm Optimization (PSO) was developed and calibrated for the problem at hand. For this purpose, a database concerning compression tests of steel equal angle structural members was constructed from available resources with geometry variables such as length, width, thickness, mechanical properties of materials such as yield strength and initial imperfections (i.e. residual peak stress and initial geometric imperfections) and critical buckling load of columns. The hybrid PSOANN model was adopted because its prediction capability is higher than the traditional technique – i.e. scaled conjugate gradient (SCG). Indeed, ANN trained by PSO delivered better performance in terms of RMSE, MAE, ErrorStD, R^2^ and Slope in comparison to ANN trained by SCG, for instance. RMSE decreases from 0.141 to 0.055; MAE decreases from 0.108 to 0.042; R^2^ increases from 0.749 to 0.959, when switching from ANN alone to hybrid PSOANN, respectively. Moreover, a Partial Dependence (PD) investigation was performed to interpret the “black-box” PSOANN model.

## Introduction

Structural elements under compression are widely used in diverse projects due to the efficiency of exploitation: truss systems^1^ or reinforced concrete columns^2–4^. In the case of instability of structural members, there is a significant risk of damage to the structure, see^2^ for an example of buckling damage of the steel bar in a reinforced concrete column. Iasinski’s work^5^ and Johnson’s formula^6^ enabled us to look at with critical loads for structural elements with low and medium slenderness. However, these formulas apply only for isotropic, homogeneous materials and there are many assumptions about the structural elements: cross-section geometry, length, boundary conditions, concentric axial loads, lack of initial stress, etc. In reality, though, these conditions are not verified. The presence of these factors reduces the stability force-resistance of structural members under compression, compared to the original design^7–9^.

In terms of modeling, theoretical investigations mainly focus on simply supported boundary conditions^10^. Recently, with the development of numerical analysis, iterative algorithms have been widely applied to nonlinear problems such as instability in the field of computational mechanics, for instance, Arc-Length method^11–13^. These methods could be applied to the numerical finite element scheme^14^ to investigate the buckling behavior of structural members. However, there are a range of drawbacks in using finite element software including mesh scheme^15^, iteration convergence^16^, implementation of initial imperfections^17^, etc. For example, for intricate shapes, automatic mesh generation using highly skewed tetrahedral elements may create distorted or poorly-shaped elements in thin-walled structures^18^. Iterative solvers in FEA may struggle to converge, particularly for problems with non-linearities such as material plasticity, contact mechanics, or large deformations^19^. In addition, simulating pre-existing stresses in welds or formed components requires advanced techniques like importing results from thermal analysis^20^.

Recently, soft computing techniques, such as surrogate models, have begun to contribute solutions to various structural and mechanical engineering problems in a significant manner. As proved in the literature, soft computing techniques provide more reliable designs^21^, evaluations^22^ and predictions^23^ than conventional methods. For instance^24^, successfully developed a surrogate model for the design of self-compacting concrete based on various mixtures of ingredients, which could be extremely time-consuming in laboratory experiments. In another study^25^, improved the design of square concrete steel tube columns by using an artificial neural network (ANN) model to explore the nonlinear relationship between geometry, mechanical properties and axial capacity of structural elements^26^. have proposed an ANN tool for prediction and design of an orthotropic steel-deck bridge, but the study has not yet been carried out, even using professional software tools. This computational model helped the authors to evaluate many different cases that would be time-consuming, effort-intensive and costly to investigate experimentally. In addition, the work of other researchers, including^27^, has confirmed the efficiency of artificial intelligence techniques for the prediction of related mechanical engineering problems.

Within this context, the current work presents the development of a surrogate model based on the combination of Particle Swarm Optimization (PSO) and ANN to predict buckling damage to steel equal angle structural members. The hybrid PSOANN model was proposed because of its higher prediction capability than the single ANN model. The database and selection of variables are presented in Sect. 2.1, while Sect. 2.2 introduces the ANN and PSO algorithms. Section 3 presents the results and a discussion thereof, including the PSO optimization process and Partial Dependence (PD) investigation to explore the relationship between variables and output response. Finally, the dependence of the output response on input variables is shown through sensitivity analysis.

## Materials and methods

### Database and selection of variables

The selected geometry variables (length, width, thickness) were chosen because they directly influence the structural behavior under compression. These parameters are critical in defining the cross-sectional properties and slenderness ratio of steel equal angle members, which are known to significantly affect buckling performance^28^. Besides, yield strength was selected as a key material property because it defines the elastic limit of the material, directly influencing the transition to plastic buckling behavior^29^. Last but not least, initial imperfections, such as residual stress peak and initial geometric deviations, were included because they are well-documented to reduce the buckling capacity of members^30^.

**Fig. 1:**
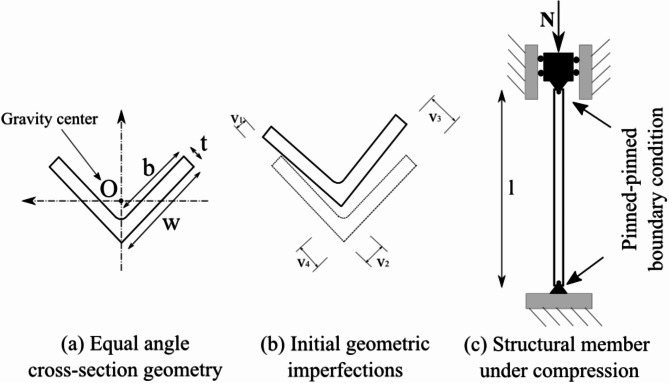
Schematization including geometry parameters for (**a**) cross-section, (**b**) initial imperfections and (**c**) testing configuration.

In this paper, 66 configurations of compression test on steel equal angle structural members were collected from the available literature^31^. In Ban et al.^31^, an experimental program was conducted to test 420 MPa high-strength steel equal angle (see Fig. 1a) columns under axial loading. The boundary conditions of the tests were pinned-pinned, as shown in Fig. 1c. The min, max, average, standard deviation, coefficient of variation and quantile values at 25 and 75% of input variables are summarized in Table 1. It should be noticed that our proposed model is valid for the range of variables shown in Table 1. High-strength steel was selected because it exhibits better mechanical behavior than normal strength steel, and therefore higher durability^32^. The main geometry parameters of the structural elements are the length l, the thickness t, the width w, the free outstanding width b of angle legs, and four initial geometric imperfections v_1_, v_2_, v_3_, v_4_ as shown in Fig. 1b. Also, the residual stress was measured and reported in the analysis^33^. The mechanical properties of the constituent materials are expressed by yield strength, also measured for each configuration, as indicated in Table 1. It should be noted that the columns were designed to exhibit slenderness ranging from 30 to 80, which covered a wide range, from medium to long columns. Further, the correlation matrix within the database is also given in Table 2, showing an initial evaluation (linear mode) of the relationship between input variables and output response.

The entire dataset was divided into training (70%) and testing (30%) subsets. Data points were randomly assigned to the training and testing sets to ensure an even distribution of variable ranges across both subsets. This randomness avoids bias toward specific parameter values in either set, therefore reduce the risk of overfitting. Moreover, to avoid bias in the training process of the ANN model, data variables were made to range from 0 to 1. This also reduces the risk of overfitting. It is interesting to notice that after training, the model’s performance was assessed on the independent testing set, which it had never used during training.

**Table 1:** Initial statistical analysis of the database used in this study.

Parameter	Notation	Unit	Role	Min	Q_25_	Q_50_	Q_75_	Max	Average	Standard deviation	Coefficient of variation (%)
Length	l	mm	Input	749.2	1194.7	1546.9	2146.8	3838	1718.2	730.38	42.51
Thickness	t	mm	Input	7.77	9.79	10.04	11.76	13.79	10.31	1.75	16.96
w/t ratio	w_t_	–	Input	11.96	14.81	15.55	15.98	16.48	15.26	1.04	6.82
b/t ratio	b_t_	–	Input	9.5	12	12.78	13.4	13.4	12.57	0.9	7.16
Yield strength	f_y_	MPa	Input	442.1	448.8	459.4	460.7	542.4	457.23	20.19	4.42
Residual stress peak	σ_r_	MPa	Input	35.93	35.93	43.75	53.86	105.77	47.47	15.01	31.62
Initial imperfection	v_1_	mm	Input	0.38	1.42	1.78	2.3	4.3	1.93	0.74	38.27
Initial imperfection	v_2_	mm	Input	0.32	0.84	1.02	1.34	2.84	1.16	0.47	41.06
Initial imperfection	v_3_	mm	Input	0.18	1.42	1.78	2.38	8.82	2.01	1.19	59
Initial imperfection	v_4_	mm	Input	0.4	0.8	1.12	1.52	2.72	1.18	0.48	40.77
Critical buckling load	N_u_	kN	Output	529.1	876.1	1081.35	1491.4	2177.2	1141.8	415.51	36.39

**Table 2:** Correlation matrix of the database used in this study.

Correlation coefficient *r*	l	t	w_t_	b_t_	f_y_	σ_*r*_	v_1_	v_2_	v_3_	v_4_	*N*_u_
l	1.00	0.19	0.01	0.02	0.28	0.04	−0.01	0.46	0.19	0.24	−0.03
t		1.00	−0.43	−0.35	0.34	0.35	−0.01	−0.02	−0.04	0.14	0.96
w_t_			1.00	0.97	−0.58	−0.95	0.32	−0.17	0.31	−0.02	−0.32
b_t_				1.00	−0.59	−0.98	0.38	−0.16	0.34	−0.01	0.25
f_y_					1.00	0.72	−0.06	0.21	0.00	0.15	0.17
σ_r_						1.00	−0.35	0.18	−0.30	0.03	0.22
v_1_							1.00	−0.10	0.49	−0.05	−0.02
v_2_			sym.					1.00	0.16	0.35	−0.17
v_3_									1.00	0.10	−0.06
v_4_										1.00	0.09
N_u_											1.00

### Methods used

#### Artificial neural network

**Fig. 2:**
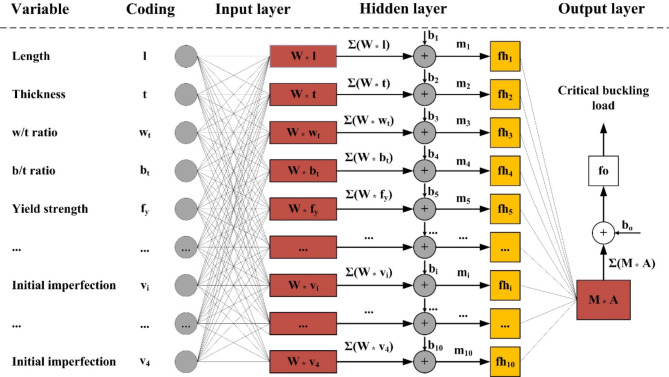
The schematization of the ANN algorithm including 10 inputs used in this study.

In this work, as the database contained a large number of input variables (i.e. 10), ANN was selected as the soft computing technique. As proved in the literature, ANN is highly efficient with large dimensional problems – for instance^25^, in structural engineering. Figure 2 shows a representation of ANN, including the input, hidden and output layers, which are three basic elements of an ANN model. The three above layers are inter-connectable via artificial neurons (i.e. computational nodes), whose objective is to compute weight parameters of the ANN model. For a single-output prediction problem, ANN model generalizes the following non-linear function:

1 $$f:X \in {{\mathbb{R}}^N} \mapsto Y \in {{\mathbb{R}}^1},$$

where *X* and *Y* represent the input and output vectors, respectively. The function *f* can be expressed as the following:

2 $$X \mapsto f\left( X \right)=fo\left( {M \times \left( {fh\left( {b+W \times X} \right)} \right)+{b_o}} \right),$$

where *W* and *M* represent the weight matrix of hidden and output layers, respectively. *fh* and *f*_*0*_ represent the activation functions of hidden and output layers, respectively. *b* and *b*_*0*_ represent the bias vectors of hidden and output layers, respectively. Such a relationship is illustrated in Fig. 2.

3 $$m=W \times X+b$$

4 $$A=fh\left( {W \times X+b} \right)$$

5 $$\lambda =fo\left( {M \times A+{b_o}} \right)$$

There are numerous algorithms available for training ANN. Some, such as Levenberg-Marquardt backpropagation, require a large amount of memory to answer numerous classification problems^34^, and the convergence rate in this approach is slow^35^. To tackle these challenges, other approaches have been developed, such as ANN models based on the principle of gradient descent^36^, i.e. the first-order conjugate gradient algorithm. However, because the line search is performed at each iteration, this strategy is still costly. In this study, we employed the scaled conjugate gradient algorithm (SCG)^36^ in conjunction with the PSO approach to train the ANN model. This type of evaluation enables us to investigate the performance of the PSO metaheuristic optimization technique.

#### Particle swarm optimization

Kennedy and Eberhart developed this efficient-swarm intelligence technique for addressing difficult optimization problems based on the social behavior of animals (e.g., a flock of birds or a school of fish)^37^. The basic idea is to move a swarm of particles iteratively to discover the global best position in a search space. The particles go across the search space, looking at various ordinary expressions for the optimum placements. The particles’ final placements are optimum answers to the challenge at hand.

**Fig. 3:**
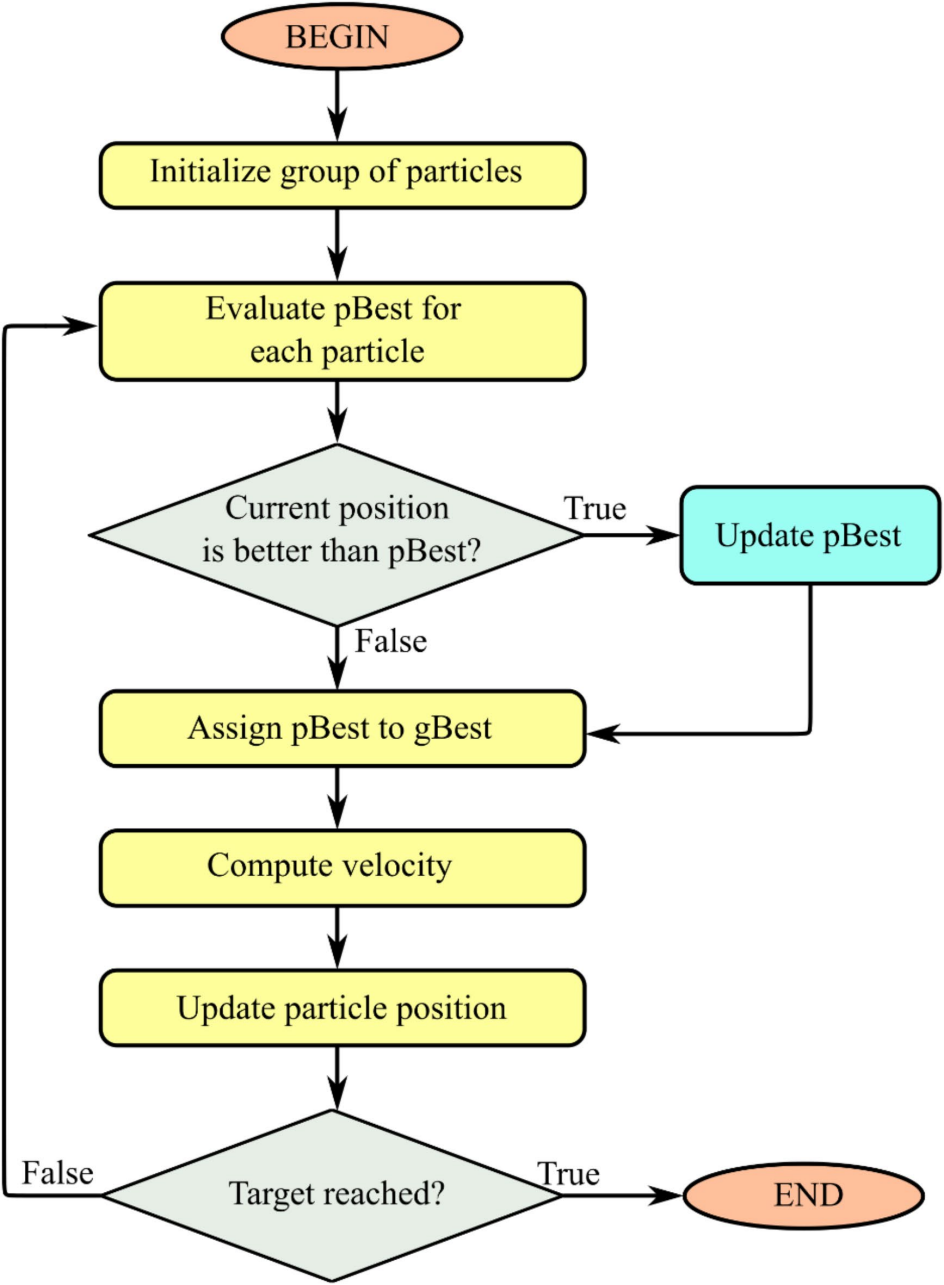
The procedure of the PSO algorithm.

Assume that the population size, or number of particles, in a *D*-dimensional search space is *m*. The particle’s location and velocity *i* (*i* = 1, 2,…, m) are represented by ${x_i}$ and ${v_i}$, respectively. Individual particles are associated with the best location in the swarm, pBest, and the best position of all particles, gBest. Figure 3 depicts the PSO method.

**Fig. 4:**
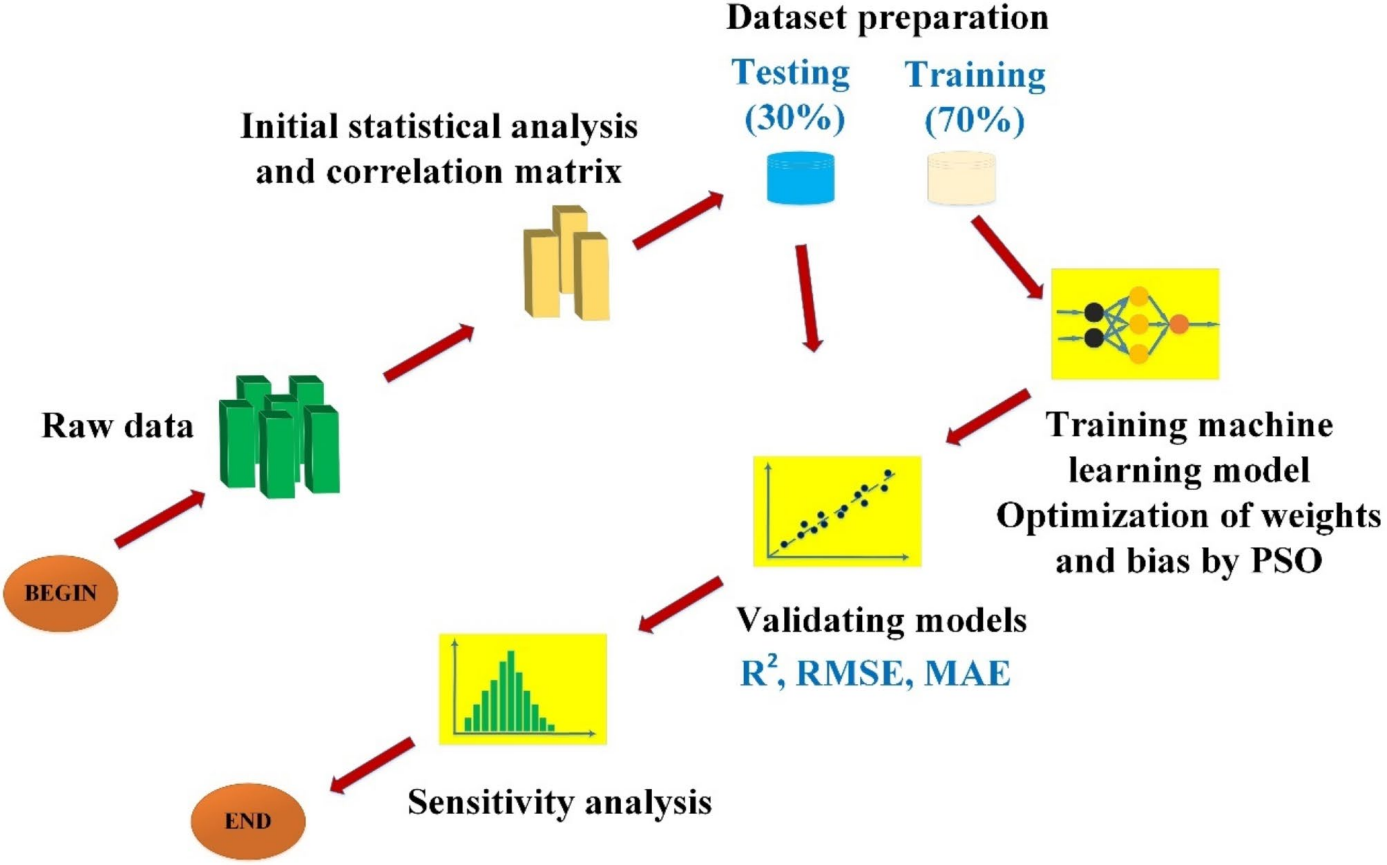
Flowchart of the current study.

Figure 4 introduces the flowchart methodology of the current study. The raw data points are analyzed statistically for deducing correlation matrix. Then the entire dataset is divided into training (70%) and testing (30%) subsets. The ANN model is trained by optimizing weights and bias using PSO global optimization technique. The model prediction performance is then validated by using different quality metrics such as R^2^, RMSE and MAE. Finally, sensitivity analysis is carried out for the proposed model and variables.

### Performance indicators

In this work, several performance indicators including the Root Mean Squared Error (RMSE), Mean Absolute Error (MAE) and coefficient of determination (R^2^) have been used to assess the quality of the prediction model, compared to experimental data. Details of these performance indicators and their formulas can be found in^38,39^.

## Results and discussion

### Optimization of ANN’s weight parameters using PSO

In this section, the weight parameters of the proposed ANN model are optimized using the PSO technique. It is worth noting that the prediction results are very much dependent on the architecture of the ANN model^40^. With a fixed quantity of inputs and outputs, the sought parameters of the ANN model are the number of hidden layers and neurons in each of them^41^. As demonstrated by many research works, one hidden layer might be enough to deal with complex non-linear relationships in the prediction problem. For example, in^42^, the authors have employed an ANN model with a single hidden layer to predict the axial capacity of square concrete-filled steel tubular columns;^43^ used such a model when investigating earthquake slope stability. For these reasons, a single-hidden-layer ANN model has been adopted in this work to reduce computational resources. In terms of number of computational nodes in each hidden layer, it has been chosen to be 2 times higher than the number of inputs, as recommended by several research works^44^. Consequently, the selected architecture of the ANN model in this study was 1 hidden layer containing 20 neurons. In terms of activation functions, a sigmoid activation function has been employed for the hidden layer and a linear activation function has been chosen for the output layer^45^. A mean square error function has been employed to be the cost function. In the current study, Matlab was employed for the training and post-processing of the model, using a Dell computer of 16 Gb of RAM and Intel Core i5, 2.9 GHz.

On the other hand, the key purpose of applying evolutionary algorithms when training AI models is to calibrate the connection between population size and the problem dimension^46^. In many cases of evolutionary algorithms – for instance, Differential Evolution – it is strongly suggested that the population should be 5 to 10 times greater than the number of predictors^47^. However, it is worth noting that a large population size does not always mean a good performance of the model^48^. In this work, the population size for PSO was chosen as 50. Inertia weight was chosen as 0.1, the personal learning coefficient was set as 2, the global learning coefficient 4 and the velocity limit 10%. It should be noted that these types of parameter ranges are often used to train AI models using PSO – for example^49^, .

**Fig. 5:**
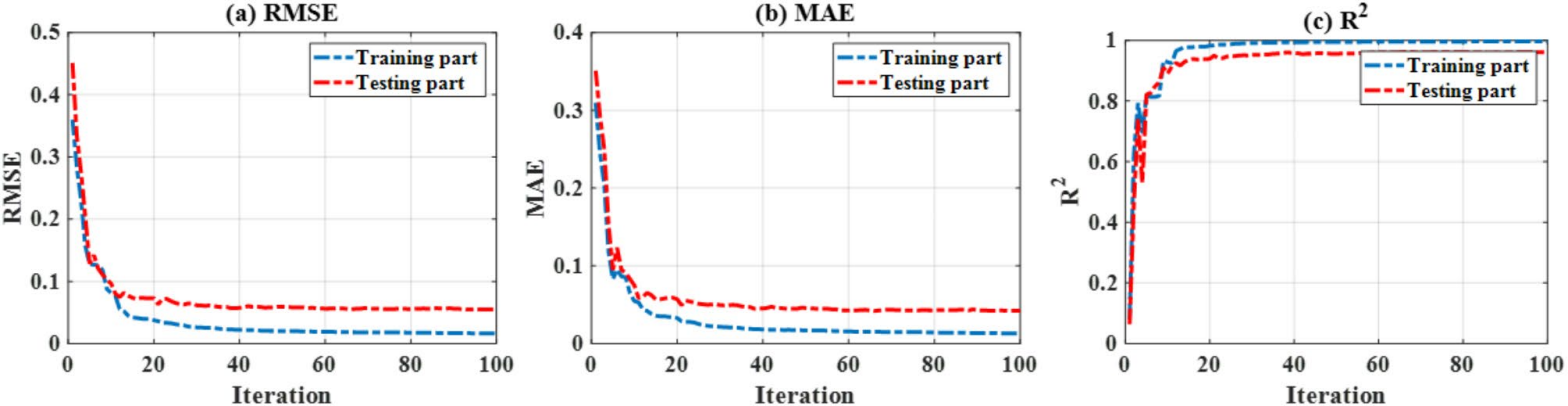
Cost functions in terms of (**a**) RMSE, (**b**) MAE and (**c**) R^2^ during the optimization of ANN’s weight parameters by using PSO, for training and testing phases, respectively.

In this work, a maximum number of 100 iterations was employed as the stopping criterion during optimization by PSO. Figure 5 presents the cost functions (RMSE, MAE and R^2^, respectively) during training and also testing. The choice of 100 iterations is shown to be relevant to obtain optimized results for all three performance indicators. During the training phase, the proposed model exhibited a good performance in terms of three quality assessments. It should be noted that the testing data were new at the time of application. This remark enables us to make the observation that no overfitting (i.e. performance indicators of testing data lead to an incorrect direction) occurred during the training phase. The robustness and efficiency of PSO during the training phase were then confirmed.

### Prediction capability

**Fig. 6:**
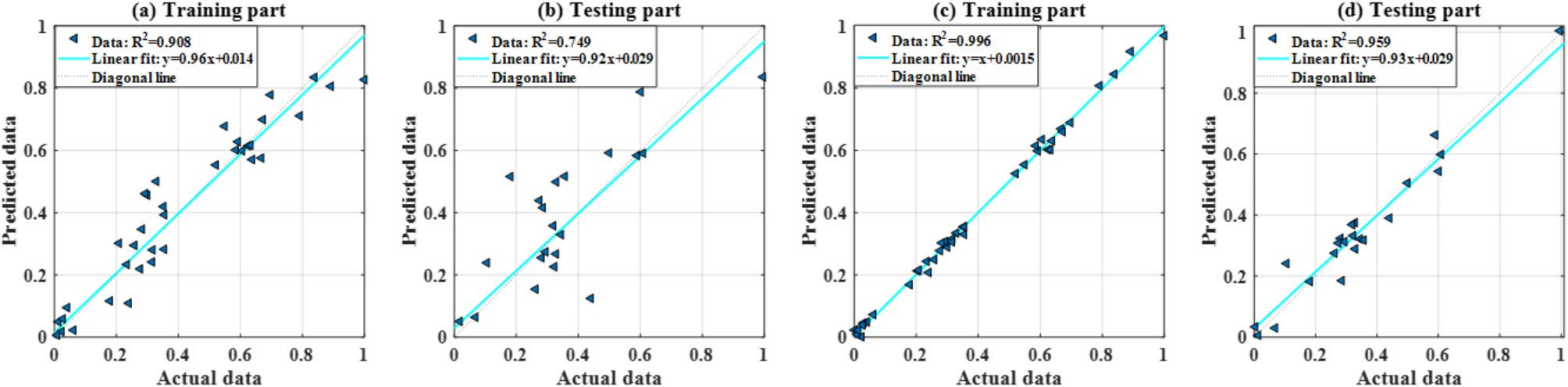
Prediction capability in terms of regression analysis using training data for (**a**) ANN alone and (**c**) PSOANN, using testing data for (**b**) ANN alone and (**d**) PSOANN.

In this section, the prediction capability of two ANN models trained by scaled conjugate gradient and PSO, respectively, is presented for comparison purposes. Figure 6 presents the expected and output data in a regression mode, when training ANN with SCG and PSO, respectively. All quantitative information was summarized in Table 3, including the values of RMSE, MAE, ErrorMean, ErrorStD, R^2^, and Slope of training and testing phases. Analysis of the results shows that for the training dataset, ANN trained by PSO yielded better performance in terms of RMSE, MAE, ErrorMean, ErrorStD, R^2^ and Slope in comparison to ANN trained by SCG. Similar results were obtained for the testing part: the PSOANN model exhibited the best prediction results for five statistical criteria: RMSE, MAE, R^2^, ErrorStD and Slope (RMSE decreases from 0.141 to 0.055; MAE decreases from 0.108 to 0.042; R^2^ increases from 0.749 to 0.959; ErrorStD decreases from 0.144 to 0.056; Slope increases from 0.922 to 0.926, using lone ANN and hybrid PSOANN, respectively). Thus, based on both error analysis and assessment of prediction quality, we can say that PSOANN is the most efficient model for predicting the buckling of a steel equal angle.

**Table 3:** The prediction capability of ANN and PSOANN.

Dataset	Model	RMSE	MAE	ErrorMean	ErrorStD	*R* ^2^	Slope
Training	ANN	0.084	0.064	0.002	0.085	0.908	0.956
	PSOANN	0.016	0.013	−0.001	0.017	0.996	0.998
Testing	ANN	0.141	0.108	−0.003	0.144	0.749	0.922
	PSOANN	0.055	0.042	−0.004	0.056	0.959	0.926

### Sensitivity analysis and discussion

In this section, PD^50^ was applied to investigate the marginal effect of input variables on the predicted result of the PSOANN prediction model, as presented in the previous section. As proved in many investigations, PD can determine the nature of the relationship between output and input (linear, monotonic or more complex)^51^. In other words, PD shows how the average prediction changes when the input is changed^52^. In this project, PD code was directly implemented in Matlab, as it is favorable for matrix computation. However, it should be noticed that PD technique has several drawbacks as below. PD assumes that the input variables being analyzed are independent of others, which may not always be true in real-world datasets where variables are often correlated. This can lead to misleading results, particularly for structural mechanics problems where geometry and material properties are interdependent. Most recently, SHAP (Shapley Additive Explanations) has received attention because this technique provides a complementary approach to PD, addressing many of its limitations^53,54^. For instance, SHAP could highlight how initial imperfections and thickness interact to affect the critical buckling load.

**Fig. 7:**
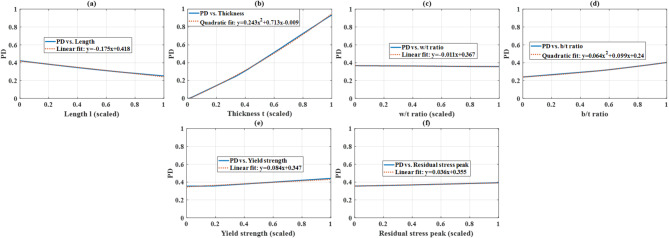
PD investigation and most appropriate fit of input variable: (**a**) length l, (**b**) thickness t, (**c**) w/t ratio, (**d**) b/t ratio, (**e**) yield strength and (**f**) residual stress peak.

**Fig. 8:**
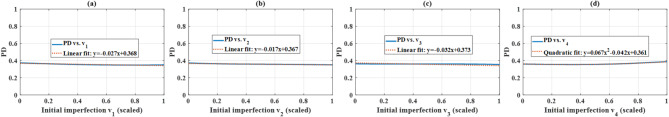
PD investigation and most appropriate fit of input variable: (**a**) initial imperfection v_1_, (**b**) initial imperfection v_2_, (**c**) initial imperfection v_3_, (**d**) initial imperfection v_4_ variable.

Based on the PSOANN model developed previously (weights and bias), PD allows us to interpret the “black-box” PSOANN model as shown in Figs. 7 and 8, respectively. Figure 7a shows that the relationship between output and length l can be fitted by a linear equation, with a negative slope of −0.175. That means the buckling capacity of the columns decreases with increasing column length. Figure 7b presents the relationship between output and thickness t. However, a nonlinear positive effect is obtained, i.e. the buckling capacity increases with increasing thickness, following a quadratic equation. It is also seen that within the range of thickness values considered in this study, the variation of PD is the highest. For other cases of b/t ratio, yield strength, residual peak stress, and initial imperfection v_4_, a positive effect was obtained. However, a negative effect was observed in the rest of the cases – i.e. w/t ratio, initial imperfections v_1_, v_2_ and v_3_. Nonetheless, the variation of PD in the case of w/t ratio and five initial imperfections was not huge compared to the cases of length, thickness and b/t ratio, for instance.

**Fig. 9:**
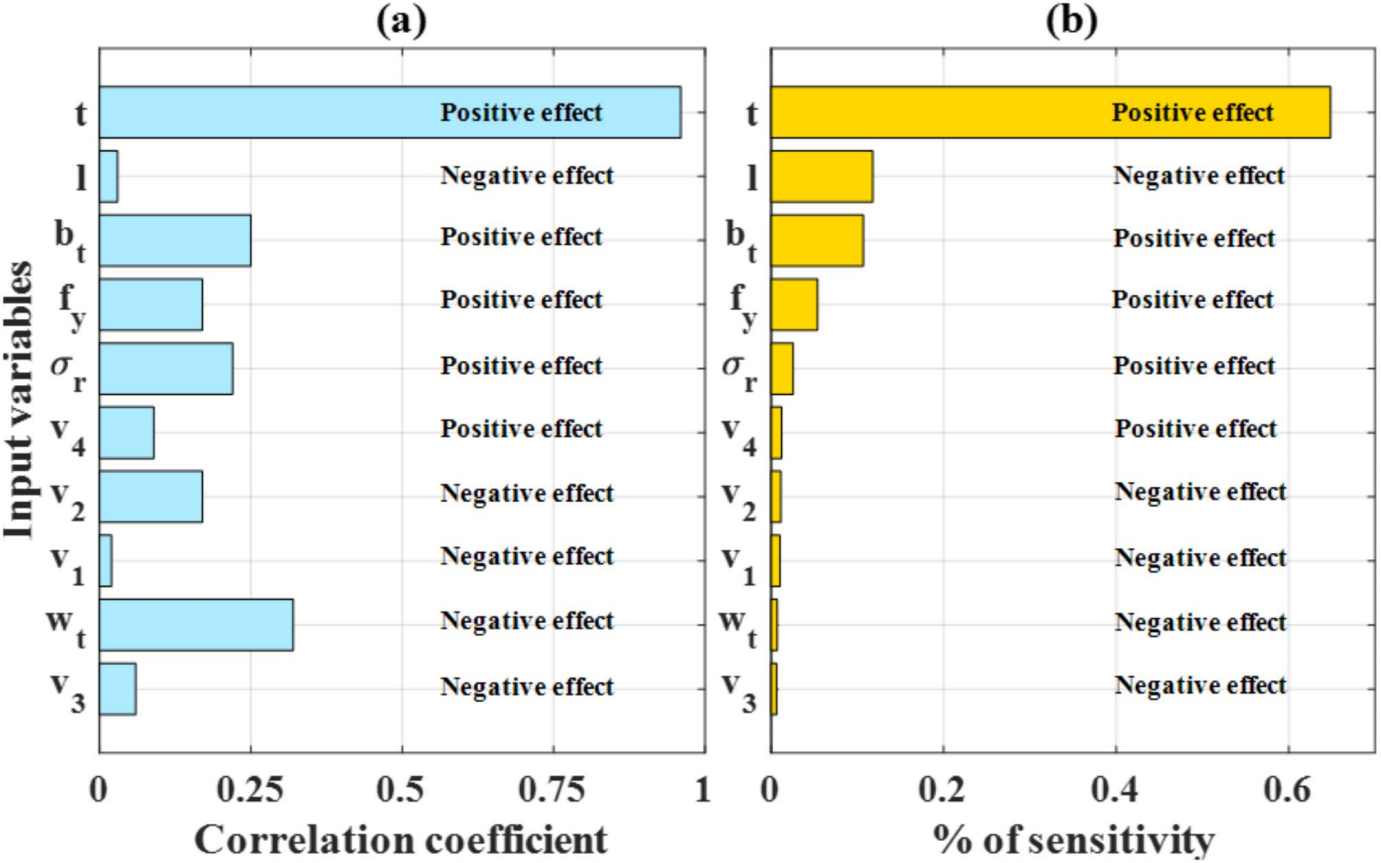
Percentage of sensitivity on input variable compared to linear correlation coefficient (direct from the database), as well as positive and negative effect of each input.

The percentage of sensitivity (i.e. level of influence) of an input variable is calculated as the integral of its respective PD curve. Ten obtained values of the area were scaled into the range of [0, 1], sorted and plotted in Fig. 9b in a bar graph, together with the linear correlation coefficient obtained directly from the database shown in Fig. 9a (also see Table 2). Thickness exhibits the most influence (positive effect) on the buckling capacity of steel equal angles, as identified by both the linear correlation coefficient and PSOANN model (through PD investigation). We can also see that the influence of thickness by far surpasses the contribution of other inputs.

As obtained by PD for the PSOANN model, l, b_t_ and f_y_ are the variables that had most influence on the buckling capacity of steel equal angles. However, a maximum of 12% sensitivity was observed for the case of length l. On the other hand, b_t_, w_t_, f_y_, σ_r_ and v_2_ exhibit linear correlation coefficients from 0.17 to 0.25, which are not relevant, especially in view of the number of entries in the database. Nonetheless, PSOANN and linear correlation coefficient are in close agreement in demonstrating the effect of input variables on the buckling capacity of columns.

The integration of the model’s predictions into current design codes or standards depends on several factors, including the model’s validation, its alignment with regulatory requirements, and the comprehensiveness of the underlying data. It should be noticed that design codes typically require extensive real-world validation across diverse scenarios to ensure reliability and consistency. Besides, the model proposed in the present study was trained on a specific dataset, which may not cover all possible structural configurations, loading conditions, or material types. For integration, these variables must align with the formats and parameters used in design codes (e.g., safety factors, design equations).

In short, the present study introduces a hybrid PSOANN model, where PSO significantly improves the training process by avoiding local minima and enhancing model performance metrics like RMSE and R^2^, as shown in Fig. 6; Table 3. The application of PSO provides a robust global optimization approach compared to single ANN model. Moreover, unlike several techniques such as Scaled Conjugate Gradient, PSO searches the solution space globally, ensuring better model generalization. Next, by performing PD analysis, the current work reveals the influence of individual input variables on the buckling load prediction. This step enhances user confidence in deploying machine learning models for structural design and assessment.

## Conclusions

In this work, an ANN model whose weights and bias are optimized by PSO is proposed to the critical buckling load of steel equal-angle structural members. Such a model was demonstrated to be efficient when compared to ANN trained by traditional techniques such as SCG. The hybrid PSOANN was a potential surrogate model for the estimation of the buckling capacity of columns, reducing cost and time in laboratory experiments. In addition, PSOANN model allows us to investigate the dependence of output on input variables by using PD. This information can be helpful in structural engineering; for example, some materials whose properties or presence in the structure do not significantly influence the performance of the final design, such as buckling resistance, strength, or durability, can be minimized to reduce production cost. For instance, reducing the need for additional coatings, reinforcements, or filler materials can speed up fabrication and reduce the cost of additional processing or handling.

Despite its achievements, the study has some limitations. First, the dataset used for training may not represent all possible geometric and material configurations. Additionally, the proposed Machine learning model poses challenges for integration into design codes and engineering practices that prioritize simplicity and transparency. Furthermore, the computational intensity of the PSOANN framework may limit its applicability for real-time or large-scale applications without further optimization.

In future works, a larger database should be investigated (to cover a broader range of values). Nonetheless, the feasibility of using surrogate modeling (e.g. ANN-based model) to study nonlinear buckling of columns should also be evaluated, together with other types of damage of structural members. Besides, SHAP analysis should be employed for further investigation of the interaction between variables. Finally, a statistical context should be applied in subsequent studies to exploit the dependence of prediction output on input variables.

## Data Availability

The datasets generated during and/or analyzed during the current study are available from the corresponding author on reasonable request.
